# Modulating Mimetic Preference with Theta Burst Stimulation of the Inferior Parietal Cortex

**DOI:** 10.3389/fpsyg.2017.02101

**Published:** 2017-12-01

**Authors:** Luca F. Ticini, Cosimo Urgesi, Sonja A. Kotz

**Affiliations:** ^1^Division of Neuroscience and Experimental Psychology, Faculty of Biological, Medical and Health Sciences, School of Biological Sciences, University of Manchester, Manchester, United Kingdom; ^2^Department of Languages and Literatures, Communication, Education and Society, University of Udine, Udine, Italy; ^3^Istituto di Ricovero e Cura a Carattere Scientifico “Eugenio Medea”, Bosisio Parini, Italy; ^4^Department of Neuropsychology, Max Planck Institute for Human Cognitive and Brain Sciences, Leipzig, Germany; ^5^Department of Neuropsychology and Psychopharmacology, Faculty of Psychology and Neuroscience, Maastricht University, Maastricht, Netherlands

**Keywords:** action, observation, preference, tools, inferior parietal lobule, cTBS, objects, affective judgments

## Abstract

We like an object more when we see someone else reaching for it. To what extent is action observation causally linked to object valuation? In this study, we set out to answer to this question by applying continuous theta burst stimulation (cTBS) over the left inferior parietal lobule (IPL). Previous studies pointed to this region as critical in the representation of others' actions and in tool manipulation. However, it is unclear to what extent IPL's involvement simply reflects action observation, rather than a casual role in objects' valuation. To clarify this issue, we measured cTBS-dependent modulations of participants' “mimetic preference ratings”, i.e., the difference between the ratings of pairs of familiar objects that were (vs. were not) reached out for by other individuals. Our result shows that cTBS increased mimetic preference ratings for tools, when compared to a control condition without stimulation. This effect was selective for items that were reached for or manipulated by another individual, whilst it was not detected in non-tool objects. Although preliminary, this finding suggests that the automatic and covert simulation of an observed action, even when there is no intention to act on an object, influences explicit affective judgments for objects. This work supports embodied cognition theories by substantiating that our subjective preference is grounded in action.

## Introduction

In the last decades, research has shown that people commonly prefer objects that are the goal of others' actions, regardless of the objects' actual value(s) or their intrinsic properties (Gollwitzer and Moskowitz, 1996). For instance, on the playground children run after the same toy even when similar toys are readily available. In social cognition, this behavior has been termed mimetic desire (Girard, 1988) and it is recognized as a case of goal contagion (Aarts et al., 2004), for which objects that are the target of another individual tend to become the goal for the observer. A functional neuroimaging study (Lebreton et al., 2012), designed to uncover the neural mechanisms of this phenomenon, surprisingly found that object preference is influenced by the activity of motor-related areas, namely the ventral premotor and the inferior parietal cortices, belonging to the action observation–action execution network (which is active during motor act execution as well as the observation of motor acts performed by others; Rizzolatti and Craighero, 2004; Fabbri-Destro and Rizzolatti, 2008; Avenanti and Urgesi, 2011). In particular, the authors found that during the observation of goal-directed actions the activity of these parietofrontal areas modulated that of the ventral valuation system where the perceived value of objects is encoded (Rangel et al., 2008; Chib et al., 2009; Lebreton et al., 2009; Peters and Büchel, 2010). However, as the correlational nature of neuroimaging cannot provide a direct causal link between brain and function, it is an open question whether this activity is merely an epiphenomenon of action perception or whether it truly reflects a neurocomputational process functionally relevant for the expression of subjective preference for a particular object. The present experiment represents a preliminary approach aimed at deciding between these two alternatives.

In healthy participants, we used offline continuous theta burst stimulation (cTBS) to interfere with the neural activity of the left inferior parietal lobule (IPL). Among the numerous TMS stimulation protocols available (for a review, see Fitzgerald et al., 2006), we used cTBS as it is known to induce, after a short and tolerable stimulation (40 s), prolonged neural inhibition (>45 min; Huang et al., 2005; Franca et al., 2006) at stimulated loci, enough to last throughout our experimental sessions. The neuronal mechanisms related to the inhibitory (or excitatory) effects of brain stimulation are not fully understood, and they vary according to the type of stimulation (i.e., frequency, intensity, number of pulses). For instance, in the case of theta burst stimulation, previous investigations reported that the application of brief bursts resulted in a transient increase in cortical facilitation (Huang and Rothwell, 2004) whilst prolonged stimulation had the opposite effect (Di Lazzaro et al., 2005; Huang et al., 2005).

We targeted the IPL for two main reasons. First, in their imaging study, Lebreton and colleagues found that its activation correlated with an object's likability and represented the first node involved in translating action observation into preference (Lebreton et al., 2012). Second, IPL's activity codes for the agent–object relationship (reviewed in Gentilucci and Volta, 2008; Fernandino and Iacoboni, 2010). In other words, the IPL holds a higher-order representation of the goal of an observed action independently of the effector (e.g., hand or mouth) used to achieve it (Jastorff et al., 2010). On the contrary, activity in the ventral premotor cortex (i.e., the other node of the action observation–action execution network involved in mimetic desires) appears more clustered around the effector (e.g., specific muscle, joint, and digit movements) performing a motor act (Jastorff et al., 2010). Stimulation of the ventral premotor cortex is also more distressing as it activates superficial nerves and muscles in the “temporalis” muscle fascia.

In addition, it is well-established that the IPL has a functional role as an interface between perceptual and motor information. Indeed, it is pivotal in visuomotor transformations (Goodale and Milner, 1992; Jeannerod et al., 1995) converting the intrinsic properties (size and shape) of an object into a pattern of goal-directed movements to reach it such as grasping (Blakemore and Sirigu, 2003; Fogassi and Luppino, 2005) and translating visual information into motor programs to achieve imitations (Mühlau et al., 2005; Molenberghs et al., 2009). Further neuroimaging (Buccino et al., 2001; Chong et al., 2008; Jastorff et al., 2010), TMS (Cattaneo et al., 2010; Jacquet and Avenanti, 2015; for a review see Avenanti et al., 2013b), and lesion studies (e.g., Buxbaum et al., 2005; Kalénine et al., 2010; for a review see Urgesi et al., 2014) showed that the IPL is implicated in the representation of goals of observed actions (Fogassi et al., 2005) and in the internal simulation of an observed motor act (i.e., motor resonance; Gallese et al., 1996; Grafton, 2009) thus demonstrating its pivotal role in goal contagion. TMS (e.g., Ishibashi et al., 2011) and neuroimaging (Kellenbach et al., 2003; Boronat et al., 2005; Canessa et al., 2008) findings implicated the left IPL also in storing motor representations for using familiar tools, in accordance with the fact that damage in this area determines toll-use deficits as shown by apraxic patients (Buxbaum et al., 2000; Rosci et al., 2003).

We chose to stimulate the left IPL for two reasons. First, because IPL's activity is generally stronger in the left hemisphere (see Peeters et al., 2009; Jastorff et al., 2010). Second, because in our experiment the majority of the observed motor acts were carried out with the contralateral right hand (see Methods; research has shown hemispheric preference for contralateral effector movements, see Pelphrey et al., 2004; Shmuelof and Zohary, 2006).

Grounding our experiment on the evidence listed above, we hypothesized that targeting the IPL with cTBS would demonstrate the casual involvement of action perception in an objects' preference. We therefore looked for variations in mimetic preference ratings (obtained comparing participants' ratings of objects that were, vs. were not, reached for by others) for familiar tools and non-tool objects, when cTBS was (or was not) applied. Our prediction was that cTBS would interfere with covert simulation of observed actions, particularly those carried out on tools, and in turn this would reduce mimetic preferences for these items. Such result would support the existence of a causal link between one's motor experience and preferences for non-valence stimuli (i.e., without an inherent emotional content). It would also support embodied cognition theories (for a review, see Ping et al., 2009) in social behavior (Aarts et al., 2004) as well as in aesthetics (Freedberg and Gallese, 2007; Cross et al., 2011; Cross and Ticini, 2012; Ticini et al., 2014) postulating that subjective preference is influenced by others' behavior.

## Methods

### Participants and experimental protocol

This study was carried out in accordance with the recommendations of the Ethics committee of the Scientific Institute (IRCCS) Eugenio Medea with written informed consent from all subjects, in accordance with the Declaration of Helsinki. The experiment was carried out on 12 volunteers (aged 22.7 ± 4.6 years; four females; 11 right-handed and one mixed-handed, Briggs and Nebes, 1975), complying with what according to Sack et al. (2009) is thought to be sufficient to demonstrate a behavioral effect in case of TMS based on group Talairach coordinates.

**Figure 1:**
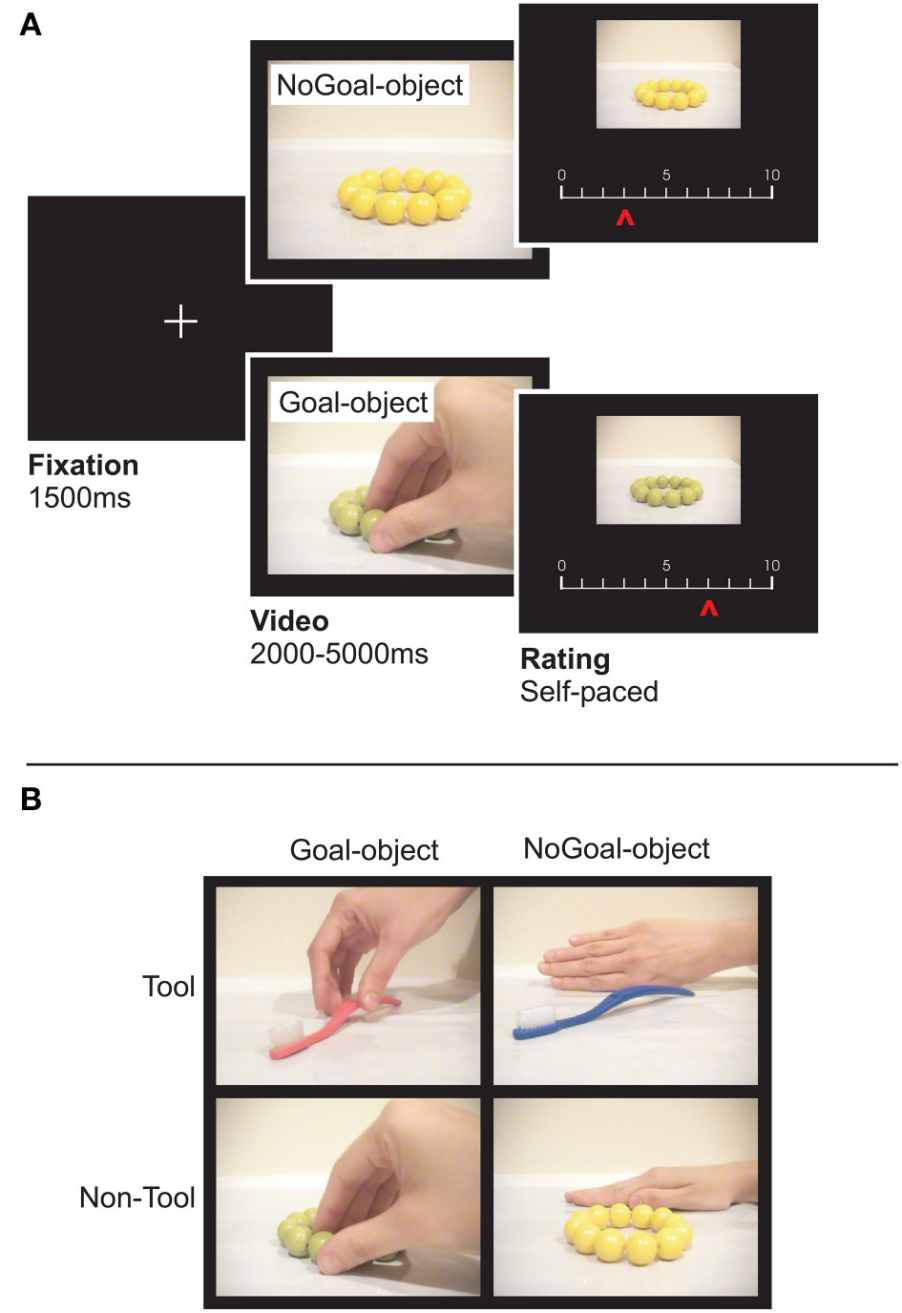
The preference-rating task. **(A)** From left to right, the figure shows successive screens displayed in one trial. Participants rated the object (“How much do you like the object?”) featured in the video by moving a cursor along a scale. Each trial started with a fixation cross followed by a video in which an item was (Goal-objects) or was not (NoGoal-objects) the target of another person's action. Then the preference scale appeared below the picture of the object to be rated (without a human agent). Two versions of identical objects, differently colored (counterbalanced across participants) were used to eliminate potential effects of color preferences at the group level. **(B)** Snapshots from the videos with Toll and Non-Tool objects. In the illustrated example, in the NoGoal-object conditions, the objects were presented statically alongside a hand that did not interact with them. These and other control conditions (e.g., the object being moved by gravity) allowed avoiding the confound that participants may prefer objects more because of the presence of a human being or because of some movement in the video.

We used 120 short videos (duration: 2–5 s; 640 × 480 pixels) from Lebreton et al. (2012) categorized in familiar tools (e.g., pen, lantern, comb, soap bottle) and non-tool objects (e.g., food, toys such as cards and teddy bear, clothes, flowers). In 60 videos, the objects were reached for by another agent (“Goal-objects”) who either moved them or not (Figure 1A). To remove the potential impact of eye gaze on the participant's judgment, the face of the agent was never visible (e.g., Bayliss et al., 2006). In 70% of the videos the action was executed with the right hand, in 6.7% with the left hand, in 15% with the mouth, and in 8.3% with other effectors (e.g., nose). The other 60 videos (“NoGoal-objects”; Figure 1B) depicted the same objects but this time they were presented either statically (48% of the objects) or they were moved by a natural forces (e.g., wind or gravity with no human interaction; 20% of the objects) or a human agent was present but did not interact with the objects (32% of the objects). This allowed us to control for the confound that participants may prefer objects in Goal-objects conditions because of the presence of a human being or because of some movement in the video. The two objects within a pair (Goal- and NoGoal-objects) differed only by color and were otherwise identical. For instance, a green candy was the Goal-object for half the subjects, and the NoGoal-object for the other half. This allowed us to eliminate color preferences at the group level (cf. Lebreton et al., 2012).

Each trial started with a fixation cross (1.5 s) followed by one of the videos. After each video, the object reappeared (436 × 326 pixels) and participants were required to rate it (“How much do you like the object?”) by moving a cursor (randomized initial position) on a sliding scale with their right hand (from 0 or not liked, to 10 or highly liked). The question was focused on the object because we aimed at assessing whether observing object-directed actions increased the perceived value of the object. Moreover, previous research demonstrated that the precise formulation of the question (e.g., “How much do you like to use the object?” or “How much would you like to acquire the object?”) is not crucial (Lebreton et al., 2012). There were no time constraints and the video presentation (Goal-objects/NoGoal-objects, Tool/Non-tool stimuli) was randomized. Stimulus presentation was obtained by using the Cogent 2000 (Wellcome Trust Centre for NeuroImaging, London, UK) library of MatLab (Math-Works).

The experiment was run twice for a total of 240 videos. In one session (i.e., cTBS), participants were first positioned for the purpose of the experiment and then cTBS was administered (the experiment started immediately after cTBS). In the other (i.e., Non-cTBS), the TMS coil was never held against the participant's head. The order of the session was counterbalanced. Each of the two sessions lasted 10 min on average and both were run with the very same experimental setup and separated by an interval of 1 h. We applied cTBS over the left IPL (MNI coordinates −58, −32, 44; transformed in Talairach space for the use of the neuronavigation software SofTaxic Optic, E.M.S. s.r.l., Bologna, Italy) corresponding to the supramarginal and angular gyri as identified by Lebreton et al. (2012). cTBS consisted of trains of bursts of three pulses at 50 Hz repeated at 5 Hz and delivered uninterrupted for 40 s for a total of 600 pulses (Huang et al., 2005). It was delivered by an air-cooled figure-of-eight coil of 70 mm diameter attached to a Magstim Rapid2 stimulator, which was kept perpendicular to the underlying gyrus with the handle pointing upward and supported manually. The stimulation intensity was set at 45% of the maximal stimulator output (see for instance Stewart et al., 2001) for all participants. Exclusion criteria were the regular use of drugs or medications, any history of psychiatric or neurological disorders, and contra-indications to transcranial magnetic stimulation (e.g., pregnancy or metallic implants), all assessed by a standard questionnaire. All participants wore earplugs and no particular discomfort or other negative side effects were reported.

### Analysis

We first conducted an omnibus repeated measured ANOVA, after having reduced the effect of inter-subject variability by normalizing (*z* scores) the preference ratings for each participants. We used “Stimulation” (cTBS, Non-cTBS) and “Action” (Goal, NoGoal-object) and “Object type” (Tool, Non-tool) as within subjects factors. Additionally, we computed the mimetic preference ratings (see Lebreton et al., 2012) as the difference in the original ratings between Goal vs. NoGoal conditions (preference for Goal-objects—preference for NoGoal-objects). This excluded effects associated with variations in preference scores among objects, as we postulated that the objects were non-valenced stimuli (i.e., without an inherent emotional content). We then used one-sample Bayesian *t*-tests to test the hypothesis that the population means for the mimetic preference in the four conditions (cTBS and No-cTBS for Tools and Non-tool objects) was not equal to zero. Bayesian *t*-tests (JASP; Love et al., 2015) employed default priors to estimate the Bayes Factors (BF; Rouder et al., 2012) and we used BF_10_ as it allows estimating the likelihood that the data fit a specified alternative hypothesis relative to the hypothesis of no effect (Rouder et al., 2012). A BF_10_ greater than 3 indicates substantial evidence for the alternative hypothesis (Wetzels and Wagenmakers, 2012), i.e., that the observed data favor the alternative hypothesis over the null hypothesis by a ratio of 3:1.

## Results

The results of the ANOVA identified a statistically significant main effect of “Action” [*F*_(1, 11)_ = 14.74, *p* = 0.003, $\eta_{\text{p}}^{2}$ = 0.57] and a significant interaction between “Stimulation,” “Action,” and “Object type” [*F*_(1, 11)_ = 5.1, *p* = 0.045, $\eta_{\text{p}}^{2}$ = 0.32]. Non-specific effects of rTMS on the ratings [“Stimulation”: *F*_(1, 11)_ = 1.3, *p* = 0.28, $\eta_{\text{p}}^{2}$ = 0.1] and other factors or interaction (*F*s < 2.74, *p*s > 0.12) were absent. We then broke the ANOVA for the factor “Object type” and run selected tests comparing the effects of stimulation within each “Object type” category. For the Tools category, we found a significant main effect of “Action” [*F*_(1, 11)_ = 23.55, *p* < 0.001, $\eta_{\text{p}}^{2}$ = 0.68] and a significant interaction between “Stimulation” and “Action” [*F*_(1, 11)_ = 7.9, *p* = 0.017, $\eta_{\text{p}}^{2}$ = 0.42]. Bonferroni-corrected *post-hoc* tests showed that the preference for Goal-objects was larger (*p*s < 0.001) when compared to NoGoal-objects in both cTBS (Goal-objects: 0.108 ± 0.054; NoGoal-objects: −0.211 ± 0.040; means ± S.E) and Non-cTBS conditions (Goal-objects: 0.024 ± 0.066; NoGoal-objects: −0.196 ± 0.056). Importantly, the preference scores between Goal-objects in cTBS were higher than those in Non-cTBS (*p* = 0.036). This indicated that cTBS selectively increased the preference for Goal-objects rather than decreasing that for NoGoal-objects. Instead, the means for NoGoal-objects did not differ between the two stimulation conditions (*p* > 0.99). As far as the Non-tools category is concerned, we found a significant main effect of “Action” [*F*_(1, 11)_ = 8.48, *p* = 0.014, $\eta_{\text{p}}^{2}$ = 0.43] confirming that in both cTBS and Non-cTBS conditions Goal-objects were preferred more than NoGoal-objects. No other results were significant (*F*s < 2.8, *p*s > 0.12). A further omnibus ANOVA conducted on the judgements' response times revealed only a significant main effect of “Object type” [*F*_(1, 11)_ = 5.17, *p* = 0.044, $\eta_{\text{p}}^{2}$ = 0.32]: the time required to express the preference for Tools (1968.38 ± 203.04; ms ± S.E.) was longer than that for Non-tools objects (1931.97 ± 155.20). Other main effects and interactions were not significant (*F*s < 1.96, *p*s > 0.19).

**Figure 2:**
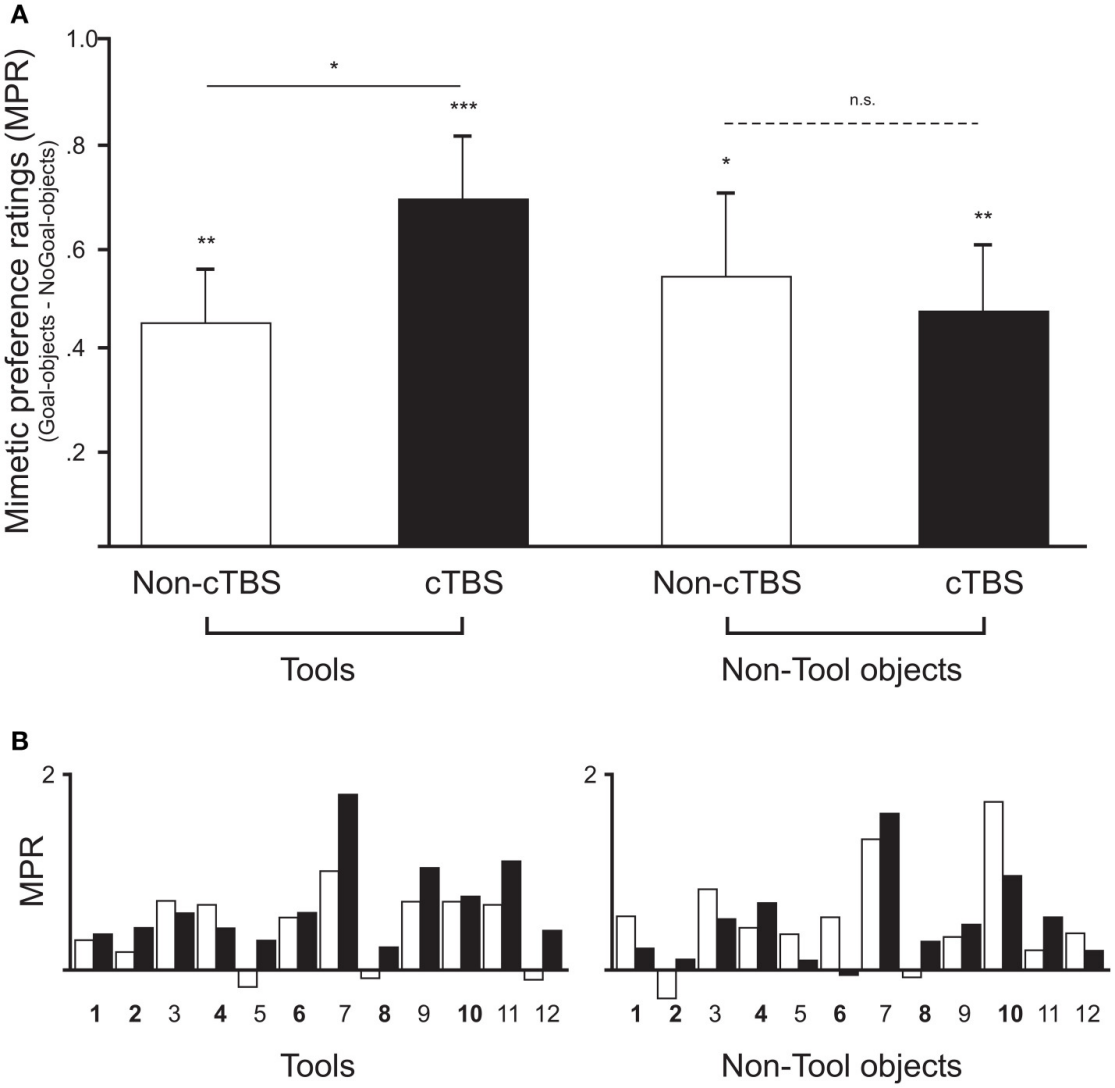
**(A)** Comparison of mimetic preference ratings (difference in preference ratings between Goal- and NoGoal-objects) across participants for Tool and Non-Tools objects in the two stimulation conditions. Empty and filled bars represent mimetic preferences for the same objects in the Non-cTBS and cTBS session, respectively. Mean mimetic preference ratings were significantly positive in all conditions (means ± standard errors of the mean; ^*^p < 0.05, ^**^p < 0.01, ^***^p < 0.001). Two-sample *t*-tests (two-tailed) and Bayesian analyses indicated a statistically significant difference between Non-cTBS and cTBS in the Tools category (solid line), whereas no significant difference was found for Non-tool objects (dashed line). **(B)** Results for each individual (numbered along the x-axis) are shown along with the counterbalancing order of stimulation (in bold participants who received cTBS first).

Table 1 shows that all mean values of mimetic preference ratings (preference for Goal-objects—preference for NoGoal-objects) were significantly larger than the test value of zero (see Figure 2). This result was expected as previous experimental evidence (Lebreton et al., 2012) indicated higher preference for Goal-objects when compared to NoGoal-objects. Notably, the BF for Tools after cTBS was very large and indicated that the data favored the one-sided alternative hypothesis that the population mean was larger than the test value of zero by a ratio of 104.2: 1. One-way ANOVAs calculated in each condition (i.e., Tools Non-cTBS, Tools cTBS, etc.) indicated that the counterbalanced order of stimulation (i.e., whether participants begun the experiment with cTBS or Non-cTBS) did not influence the results (*F*s < 5.6; *p*s > 0.16).

**Table 1:** Values of mimetic preference ratings measured across conditions.

**Conditions**	**Mean**	**S.E**.	***t*-values**	***p*-values (two-tailed)**	**BF_10_**	**C.I**.		**Cohen's d**
Tools Non-cTBS	0.44	0.11	3.9	0.002	**18.9**	0.19	0.69	1.13
Tools cTBS	0.68	0.13	5.1	<0.001	**104.2**	0.39	0.98	1.48
Non-tools Non-cTBS	0.53	0.16	3.3	0.008	**7.4**	0.17	0.88	0.94
Non-tools cTBS	0.47	0.13	3.6	0.004	**12.6**	0.19	0.76	1.05

*S.E., Standard error; BF, Bayes factor; C.I., Confidence interval*.

*The results of the statistical tests contrasting the means against the test value of zero are shown*.

A two-sample *t*-test (two-tailed) further showed that the mimetic preference for Tools after cTBS was significantly higher than that for Tools in the Non-cTBS conditions [*t*_(11)_ = 2.8, *p* = 0.017, d = 0.81, C.I. 0.05 to 0.43]. The BF confirmed these results by indicating that the data favored the alternative hypothesis that the mimetic preference mean was higher in cTBS than in Non-cTBS by a ratio of 3.8: 1. This was clearly not the case for Non-tool objects [*t*_(11)_ = −0.48, *p* = 0.64, d = −0.14; BF_10_ = 0.3; C.I. −0.31 to 0.19].

## Discussion

In this study, we used non-invasive brain stimulation to test the embodied cognition's hypothesis that subjective preference is grounded in action, while we recorded the preference of healthy volunteers for objects that were (or were not) reached for or manipulated by other individuals. The result indicates that IPL stimulation, which is known to interfere with processing goal-related information (Cattaneo et al., 2010; Puzzo et al., 2013; for a review see Avenanti et al., 2013b) and tool manipulation (Ishibashi et al., 2011), led to a significant increase in mimetic preference ratings of tools. No effects were found on mimetic preferences of other familiar objects. We argue that, albeit preliminary, this result suggests a left IPL's contribution in integrating the representation of others' actions (Iacoboni et al., 1999; Grèzes et al., 2003; Johnson-Frey et al., 2003) with subjective preference for objects (Lebreton et al., 2009). We believe that the absence of an effect of the order of stimulation indicates that cTBS was not particularly disturbing *per se* (as people may rely more on others' choice in distressing situations) and did not induce arousal for objects (e.g., Dräger et al., 2004).

The present outcome prompts to question of why IPL's stimulation increased mimetic preference rather than the opposite, as we predicted. Indeed, previous research indicated that prolonged cTBS results in transient inhibitory effects (for a review, see Fitzgerald et al., 2006; Parkin et al., 2015). For instance, it determines a reduction in cortical excitability (or amplitude of motor-evoked potentials) when a testing single pulse of transcranial magnetic stimulation is applied over the primary motor cortex (Di Lazzaro et al., 2005; Huang et al., 2005). Although the mechanisms through which cTBS interferes with brain activity are not completely clear (Fitzgerald et al., 2006), some authors suggest that the change produced by cTBS may be related to a decreased effectiveness of synaptic connections recruited in the circuits that are involved in the generation of motor-evoked potentials (Huang et al., 2005).

In spite of the fact that some deemed irrelevant the direction of the behavioral effects when using TMS to empirically test the causal role of a brain area (Silvanto et al., 2008), two alternative explanations of our result are possible. On the one hand, cTBS may have increased the functional activation of the stimulated IPL (Siebner et al., 2001), thus permitting a better embodiment of the observed actions. In this regard, an interesting report indicated that interferential TMS over IPL improved recognition of emotional body movements (i.e., emotional expressions; Engelen et al., 2015). On the other, cTBS suppression of the IPL may have triggered compensatory activity in other sensorimotor areas with the result of facilitating the brain response to observed actions (e.g., Ubaldi et al., 2015). This apparently “paradoxical” facilitatory effect has been predicted and reported before (Kilner et al., 2007; Gazzola and Keysers, 2009; Friston et al., 2011; Schippers and Keysers, 2011; D'Ausilio et al., 2012; Arfeller et al., 2013; Avenanti et al., 2013a). In other words, disruptive cTBS over IPL may have increased motor resonance in frontal regions (Ubaldi et al., 2015) and in so doing facilitated the brain response to action observation (e.g., Avenanti et al., 2013a). In turn, as proposed by Lebreton et al. (2012), it is plausible that the increased activity in frontal areas would have engaged the ventral valuation system encoding the perceived value of objects (Rangel et al., 2008; Chib et al., 2009; Lebreton et al., 2009; Peters and Büchel, 2010). Obviously, the question of what are the underlying mechanisms associated to the spread of cTBS effects cannot be address in the current study as they would require, for instance, a combination of TMS and fMRI data in order to visualize compensatory processes. Another caveat (discussed below) is the absence of a control site. Overall, whether cTBS determined an increase or decrease of brain activity, our result seems to indicate that IPL belongs to a neural mechanism that translate the observation of goal-directed actions into preference ratings. We further believe that the pattern of our results rules out the possibility that the increase in mimetic preference toward tools was due to perceptual ease or fluency. This is observed when previous experience interacting with items affects one's own preference toward them (for a review, see Reber et al., 2004). Moreover, as the counterbalanced order of stimulation doesn't affect the pattern of results (see Results and Figure 2B), we can also exclude that our measurements were affected by behavioral facilitation. This is observed when the modulation of baseline neural activity though adaptation to a stimulus leads to state-dependency TMS effects, i.e., the stimulation of functionally distinct neural populations within the targeted region (e.g., Cattaneo and Silvanto, 2008).

Additionally, we found that the time required to express the preference for tools was longer than for non-tool objects. This result could be explained, at least indirectly, by the fact that viewing tools automatically activates neural representations associated with their manipulation, which differ from that for other objects (Castiello, 2005; Proverbio et al., 2007, 2011, 2013; Cardellicchio et al., 2011). The integration of several complementary higher order relationships among action, affordance associated with tool manipulation, target and preference may require a longer processing time.

As mentioned above, a limitation of the current preliminary study is the absence of an active control stimulation site. The absence of control site means that we cannot exclude the possibility that cTBS applied elsewhere in the brain could cause similar effects on mimetic preference of tool objects. However, the choice of an “ideal” control site appears difficult when it comes to experiments requiring subjective judgements: TMS at different sites may influence participants' ratings in ways that cannot be always controlled for. For instance, TMS may cause diverse degrees of discomfort or distress associated with visual, acoustic, and tactile sensations or with activation of superficial nerves and muscles (e.g., when targeting the ventral premotor cortex through the “temporalis” muscle fascia). In addition, TMS of other sensorimotor areas (e.g., striate and extra-striate cortices, superior temporal sulcus) may interfere with the perception and processing of the visual stimuli *per se* (in our case, both Goal- and NoGoal-objects), and stimulation of prefrontal and posterior parietal cortices may modulate object likability (e.g., Cattaneo et al., 2014) independently of whether an object is the goal of an action or not. Overall, we believe that the stimulation of the IPL was meaningful to address the research question, as demonstrated by the pattern of our results, and we acknowledge the fact that future studies should include an appropriate active control site.

To conclude, our preliminary result demonstrates that an object's preference is contingent on the representation of others' behavior in the brain of the beholder. We believe that this outcome is relevant to our understanding of the neural mechanism that, through actions, allows conveying the value of items from individual to individual and automatically influence preference choices for the objects present in our environment.

## Author contributions

CU, LT, and SK conceived the work, interpreted the data, draft and revised the manuscript. LT and CU acquired the data and LT analyzed them.

### Conflict of interest statement

The authors declare that the research was conducted in the absence of any commercial or financial relationships that could be construed as a potential conflict of interest.
